# Supplementary material for: Resolving the full spectrum of human genome variation using Linked-Reads
Source: Genome Res. 2019 Apr;29(4):635–45. doi: 10.1101/gr.234443.118 (PMC6442396; doi:10.1101/gr.234443.118)
Supplement: Supplemental Material [file supp_gr.234443.118_Supplemental_longranger-2.2.zip › longranger-2.2.tar.bz2!/lr2.2-public/lib/stan/cmdstan/stan/doc/api/html/math_2matrix_2mdivide__left__tri__low_8hpp_source.html]

Stan: src/stan/math/matrix/mdivide\_left\_tri\_low.hpp Source File


|  |  |
| --- | --- |
|  | Stan  2.5.0  probability, sampling & optimization |


- Main Page
- Related Pages
- Namespaces
- Classes
- Files

- File List
- File Members

All Classes Namespaces Files Functions Variables Typedefs Enumerations Enumerator Friends Macros Pages

- src
- stan
- math
- matrix

mdivide\_left\_tri\_low.hpp

Go to the documentation of this file.

1 #ifndef STAN\_\_MATH\_\_MATRIX\_\_MDIVIDE\_LEFT\_TRI\_LOW\_HPP

2 #define STAN\_\_MATH\_\_MATRIX\_\_MDIVIDE\_LEFT\_TRI\_LOW\_HPP

3

4 #include <boost/math/tools/promotion.hpp>

5 #include <stan/math/matrix/Eigen.hpp>

6 #include <stan/math/matrix/mdivide\_left\_tri.hpp>

7 #include <stan/math/error\_handling/matrix/check\_square.hpp>

8 #include <stan/math/error\_handling/matrix/check\_multiplicable.hpp>

9 namespace stan {

10  namespace math {

11

12  template <typename T1, typename T2, int R1,int C1,int R2,int C2>

13  inline

14  Eigen::Matrix<typename boost::math::tools::promote\_args<T1,T2>::type,

15  R1,C2>

16  mdivide\_left\_tri\_low(const Eigen::Matrix<T1,R1,C1> &A,

17  const Eigen::Matrix<T2,R2,C2> &b) {

18  stan::math::check\_square("mdivide\_left\_tri\_low(%1%)",A,"A",(double\*)0);

19  stan::math::check\_multiplicable("mdivide\_left\_tri\_low(%1%)",A,"A",

20  b,"b",(double\*)0);

21  // return promote\_common<Eigen::Matrix<T1,R1,C1>,

22  // Eigen::Matrix<T2,R1,C1> >(A)

23  // .template triangularView<Eigen::Lower>()

24  // .solve( promote\_common<Eigen::Matrix<T1,R2,C2>,

25  // Eigen::Matrix<T2,R2,C2> >(b) );

26  return mdivide\_left\_tri<Eigen::Lower,T1,T2,R1,C1,R2,C2>(A,b);

27  }

28  template <typename T,int R1, int C1>

29  inline

30  Eigen::Matrix<T,R1,C1>

31  mdivide\_left\_tri\_low(const Eigen::Matrix<T,R1,C1> &A) {

32  stan::math::check\_square("mdivide\_left\_tri\_low(%1%)",A,"A",(double\*)0);

33  // int n = A.rows();

34  // Eigen::Matrix<T,Eigen::Dynamic,Eigen::Dynamic> b;

35  // b.setIdentity(n,n);

36  // A.template triangularView<Eigen::Lower>().solveInPlace(b);

37  // return b;

38  return mdivide\_left\_tri<Eigen::Lower,T,R1,C1>(A);

39  }

40

41  }

42 }

43 #endif

stan::math::mdivide\_left\_tri\_low

Eigen::Matrix< typename boost::math::tools::promote\_args< T1, T2 >::type, R1, C2 > mdivide\_left\_tri\_low(const Eigen::Matrix< T1, R1, C1 > &A, const Eigen::Matrix< T2, R2, C2 > &b)

**Definition:** mdivide\_left\_tri\_low.hpp:16

check\_multiplicable.hpp

stan::math::check\_multiplicable

bool check\_multiplicable(const char \*function, const T1 &y1, const char \*name1, const T2 &y2, const char \*name2, T\_result \*result)

**Definition:** check\_multiplicable.hpp:15

mdivide\_left\_tri.hpp

Eigen.hpp

stan::math::check\_square

bool check\_square(const char \*function, const Eigen::Matrix< T\_y, Eigen::Dynamic, Eigen::Dynamic > &y, const char \*name, T\_result \*result)

Return true if the specified matrix is square.

**Definition:** check\_square.hpp:24

check\_square.hpp


---

[ Stan Home Page ]

*© 2011–2014,
Stan Development Team.*
